# Primary transitional cell carcinoma of the fallopian tube in a premenopausal woman: A case report and review of literature

**DOI:** 10.4103/0971-5851.56335

**Published:** 2009

**Authors:** Manoj R. Babu, Altaf Gauhar Haji, K. Chitrathara, D. K. Vijaykumar, Jayeeta Samanta, K. R. Hiran

**Affiliations:** *Department of Surgical Oncology, Amrita Institute of Medical Sciences, Kochi, Kerala, India*; 1 *Institute of Reproductive Medicine, Salt Lake City, Kolkata, India*; 2 *Department of Pathology, Amrita Institute of Medical Sciences, Kochi, Kerala, India*

**Keywords:** *Carcinoma ovary*, *fallopian tube*, *transitional cell carcinoma*

## Abstract

Transitional carcinomas are extremely rare in the fallopian tube. A 41-year-old premenopausal lady presented with colicky abdominal pain and was found to have a left-sided pelvic mass on examination. In view of the elevated CA-125 and imaging findings suggestive of ovarian mass, she underwent staging laparotomy. Pathological examination confirmed a primary transitional cell carcinoma of the left fallopian tube. Review of available literature suggested that the primary transitional cell carcinoma is probably less aggressive compared to classical adenocarcinoma of the fallopian tube, and it has to be distinguished from the recently recognized entity, parafallopian tube transitional cell carcinoma.

## INTRODUCTION

Primary fallopian tube carcinoma (PFTC), first described by Renaud in 1847, is the least common gynecological malignancy encountered in practice. Though histologically and clinically, fallopian tube carcinoma resembles epithelial ovarian cancer (EOC), transitional cell carcinoma of the fallopian tube is a very rare histological variant, with only around 20 cases having been reported worldwide so far.[1,2] 

## CASE REPORT

A 41-year-old lady presented with intermittent colicky lower abdominal pain of 1-month duration. Her menstrual cycles were regular, 2-3/28 days. She had two vaginal deliveries in the past. There was no history of breast or ovarian cancer in her family. Her bladder and bowel habits were normal.

On examination, she was of average build with good performance status. Her vitals were stable, with no pallor, edema or lymphadenopathy. Abdominal palpation showed a supra-pubic mass of 12 cm diameter, arising from the pelvis, towards the left side. The mass was of variable consistency, with restricted mobility. On vaginal examination, a left adnexal mass of about 14 cm diameter, adherent to the uterus, was felt. However, per rectal examination did not reveal any mass in the Pouch of Douglas (POD).

**Figure 1:**
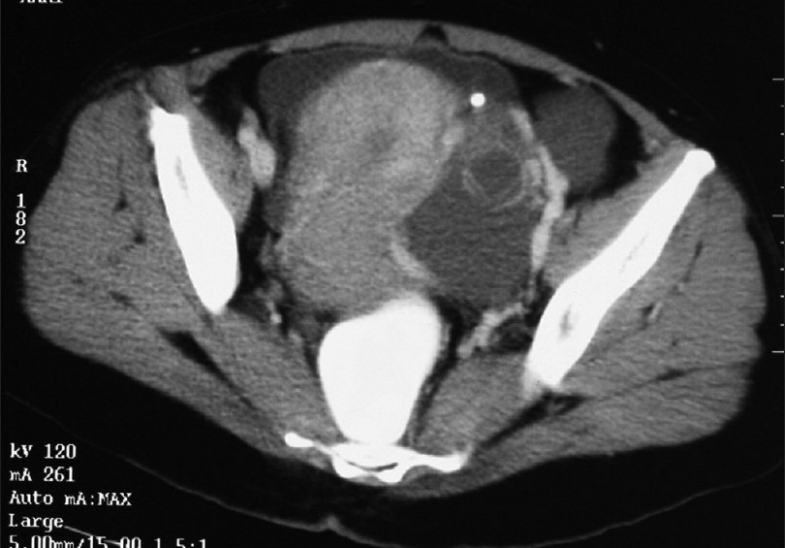
Contrast-enhanced CT scan showing tumor with increased vascularity in the periphery

Patient was evaluated with routine blood investigations, tumor markers and imaging, including ultrasound and a CT scan of the abdomen and pelvis[Figure 1]. The investigation reports are summarized in Table 1.

**Table 1:** Laboratory and imaging reports

Investigation	Result
Hemoglobin	13.2 g/dL
Random blood sugar	125 mg/dL
Liver and renal functions	Normal
CA-125	1391 IU/L (normal, <35 IU/L)
CEA	1-88 ng/mL (normal, <3 ng/mL)
Pap smear	Normal
Chest X-ray	Normal
USG abdomen and pelvis	Left adnexal mass with solid and cystic areas
CT abdomen and pelvis	A mixed-density lesion of 12 cm in the left adnexal region with enhancing solid and cystic components. Tortuous enlarged vessels noted in the periphery of the lesion. Moderate ascites. Right ovary normal. Left ovary not visualized separately. Uterus bulky with no focal lesions

**Figure 2:**
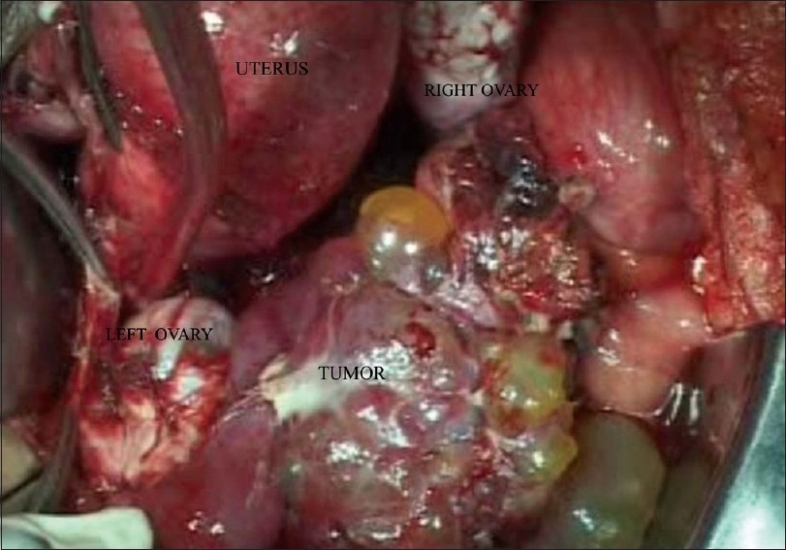
Mass arising from the left fallopian tube. Both ovaries appear free of disease

Thus, with a provisional diagnosis of ovarian malignancy, the patient underwent a staging laparotomy. Per-operatively she had about 750 mL of hemorrhagic ascitic fluid. There was an irregular mass arising from the left fallopian tube, of size 14 × 8 cm, with predominant solid areas along with cystic areas, protruding from the fimbrial opening, consisting of multiple thin-walled cysts of size 0.5 to 2 cm. Many of the cysts were already ruptured, and their serosanguineous contents were leaking into the peritoneal cavity. Both the ovaries, on gross examination, appeared apparently normal[Figure 2]. No obvious deposits were found on the POD, abdominal or diaphragmatic peritoneal surfaces. Liver and omentum appeared free of disease. No significantly enlarged pelvic or para-aortic nodes were present.

**Figure 3:**
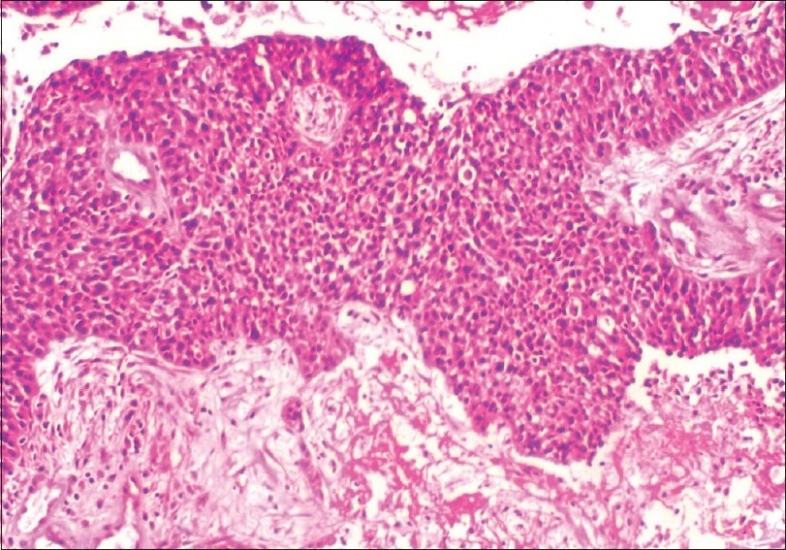
Microscopy - neoplastic cells in sheets and trabeculae. Necrotic cells can also be seen, H and E, ×100

**Figure 4:**
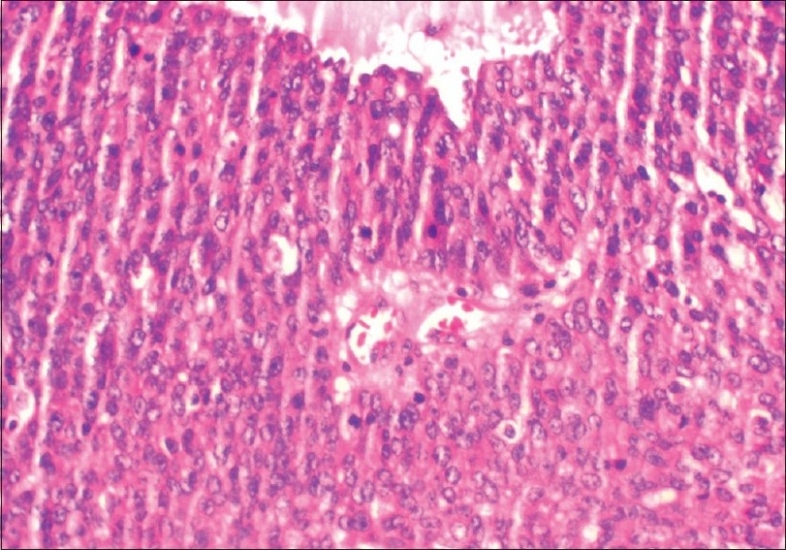
Microscopy-cells arranged around a central vascular core. Many mitotic figures are also seen, H and E, ×200

Intraoperative frozen section from the tumor was reported as malignant epithelial tumor. In view of this, a total abdominal hysterectomy, bilateral salpingo-oophorectomy, infra-colic omentectomy, appendicectomy, bilateral pelvic and para-aortic lymphadenectomy, along with multiple peritoneal biopsies, were performed. Final histopathologic examination showed transitional cell carcinoma of the left fallopian tube with marked desmoplastic reaction, papillary fronds and areas of necrosis and invasion of the muscular wall[Figures 3  and 4]. Both ovaries and uterus were free of disease. Retroperitoneal nodes, omentum and appendix showed no evidence of metastasis. Ascitic fluid cytology was negative for malignant cells. Hence a final diagnosis of transitional cell carcinoma of left fallopian tube, FIGO stage IC, was made.

Postoperatively she received 6 cycles of adjuvant chemotherapy with paclitaxel (175 mg/ m^2^) and Carboplatin (AUC – 6). At the time of completion of treatment, her CA-125 was 13.3 IU/L. She was disease-free after 1 year and remains to be so and is on regular follow-up since then. The latest CA-125 at the time of last follow-up was normal, and a CT scan of the abdomen and pelvis showed no evidence of disease.

## DISCUSSION

Fallopian tube carcinoma is often preoperatively misdiagnosed as ovarian carcinoma. Accurate diagnosis and differentiation of PFTC from lesions that have spread from the ipsilateral ovary by direct extension or from the contralateral ovary by trans-coelomic route are important for monitoring trends in incidence, for better characterization of prognostic features and possibly for improved management. Criteria for establishing the diagnosis of primary fallopian tube carcinoma, first suggested by Hu *et al.,* later modified by Sedlis[3] in 1978, include all of the following:

- The tumor arises from the endosalpinx.
- The histological pattern reproduces the epithelium of the tubal mucosa.
- Transition from benign to malignant epithelium is found.
- The ovaries are either normal or with tumor smaller than that of the tube.

PFTC is more commonly seen in postmenopausal women, but it is not clear whether the same is true about primary transitional carcinoma.

Patients with primary fallopian tube cancer appear to have a shorter history of symptoms compared to those with epithelial ovarian cancer (EOC). About 50% to 60% of patients present with vaginal bleeding or spotting, abdominal and/or pelvic mass; and 30% to 40% of patients present with colicky or dull abdominal pain.[4] Latzke triad of symptoms, consisting of intermittent profuse serosanguinous vaginal discharge, abdominal and/or pelvic pain, is reported in 15% of cases.

Between 0% and 23% of cases of PFTC may have abnormal cervical cytology suggestive of adenocarcinoma.[4] Pap smear was negative in the present case as well. There is an isolated case report of transitional cell carcinoma of the fallopian tube diagnosed after a total abdominal hysterectomy with salpingo-oophorectomy done for repeated Pap smear reports suggestive of squamous cell carcinoma.[1] 

The reported rate of preoperative diagnosis in fallopian tube carcinoma is low. Baekelandt *et al.,* have reported a preoperative diagnosis rate of 2%.[5] Both the USG and the CT scan could not suggest a diagnosis of PFTC in this case because the ovary on the left side was not seen separately from the adnexal mass.

In this case, a color Doppler was not done; but the CT scan finding of tortuous enlarged vessels, noted in the periphery of the mass with ascites, and the markedly elevated CA-125 level were strongly suggestive of malignancy.

The pretreatment CA-125 level is an independent prognostic factor of disease-free survival and overall survival (OS) in patients with PFTC. CA-125 is also found to be a good marker for post-treatment follow-up, similar to ovarian carcinoma.[4] 

Primary adenocarcinoma constitutes more than 90% of the malignant tumors of the fallopian tube. Other less common histological types include clear cell carcinoma, squamous cell carcinoma, endometrioid carcinoma, transitional cell carcinoma, mixed carcinoma and sarcoma. Transitional cell carcinoma of the fallopian tube is a very rare histological pattern of fallopian tube carcinoma. The morphology of transitional carcinoma is similar to that of tumors of the urothelium. There is a newly recognized entity known as parafallopian tube carcinoma, where the tumor is closely attached to the extraluminal portion of the tube. It is presumed to arise from Walthard's rest, paratubal cyst or directly from the tubal serosa.[6] Hence it is important to distinguish PTCC of fallopian tube from parafallopian tube transitional cell carcinoma to identify any difference in clinical characteristics.

Surgery is the treatment of choice, as in cases of ovarian tumors. A staging laparotomy through a generous midline vertical incision is recommended, as in cases of ovarian cancer. Studies suggest that patients with PFTC have higher rates of retroperitoneal and distant nodal metastases than those with epithelial ovarian cancer.[7] Hence a systematic pelvic and para-aortic lymphadenectomy is preferred to selective lymph node sampling.

Patients with stage I disease without risk factors like involvement of the muscularis layer were reported to have 100% 5-year survival and need not be treated with adjuvant chemotherapy. In contrast, stage I with invasion of the muscularis layer or tumor in the fimbria and higher stages should receive adjuvant chemotherapy.[7,8] Adjuvant chemotherapy with a combination of carboplatin and paclitaxel, which is the gold standard of chemotherapy in epithelial ovarian cancer, is now increasingly being used in PFTC.[9] Our literature search did not reveal any report of conservative management in the form of unilateral salpingectomy or salpingo-oophorectomy for early-stage PTCC of the fallopian tube.

Uehira *et al.,* in a study comparing transitional cell (TC)–predominant PFTC with non–TC-predominant PFTC found that TC-predominant tumors tended to relapse later (mean, 31.2 months after diagnosis) than non–TC-predominant tumors (mean, 14.4 months after diagnosis), resulting in a significant difference in the 2-year disease-free survival rate. Hence he concluded that TC pattern and non-TC pattern are considered to be worthy of distinction in PFTC.[10] 

## CONCLUSION

Most of our experience regarding PFTC is either extrapolation from the more common ovarian carcinoma or from isolated case series and retrospective studies over a long period of time. It appears that it tends to present earlier than EOC and has a higher incidence of lymphatic spread, necessitating routine evaluation of lymph nodes. But our knowledge of rare histological types like transitional cell carcinoma is still limited. 
